# Amendments Made to the Older Americans Act Titles III, IV, and V

**DOI:** 10.1093/geroni/igaa057.2520

**Published:** 2020-12-16

**Authors:** Lieke van Heumen

**Affiliations:** University of Illinois at Chicago, Chicago, Illinois, United States

## Abstract

This presentation discusses amendments made to the Older Americans Act titles III, IV and V through the most recent reauthorization. Title III reauthorizes Title IV programs, Title IV reauthorizes title V programs and Title V reauthorizes title VI programs. The reauthorizations each include a seven percent increase in fiscal year 2020 and a six percent increase per year for the next four fiscal years. New in title III are an amendment that allows projects that address traumatic brain injury among older adults to be included in grant programs, an amendment that improves an existing transportation grant program and an amendment that improves an existing grant program for multigenerational collaboration. Additionally, existing falls prevention and chronic disease self-management programs are codified within title III. New in title IV is an amendment that allows eligible previously incarcerated individuals to be considered a prioritized population for the Senior Community Service Employment Program.

